# 1-Year follow-up evaluation of TAK-003 dengue vaccine effectiveness against symptomatic disease and hospitalisation in adolescents during the 2024–2025 outbreak in São Paulo, Brazil: a test-negative case-control study

**DOI:** 10.1016/j.eclinm.2026.104181

**Published:** 2026-08-31

**Authors:** Lisany Krug Mareto, Thiago Sanches Brumatti, Roberto Dias de Oliveira, Patricia Vieira da Silva, Edinéia Ribeiro dos Santos, Tatiana Lang D’Agostini, Regiane A. Cardoso De Paula, Natalie E. Dean, Albert I. Ko, Derek A.T. Cummings, Jason R. Andrews, Matt D.T. Hitchings, Otavio Ranzani, Julio Croda

**Affiliations:** a School of Medicine, Federal University of Mato Grosso do Sul, Campo Grande, MS, Brazil; b Disease Control Coordination of the São Paulo State Department of Health, São Paulo, Brazil; c State University of Mato Grosso do Sul - UEMS, Dourados, MS, Brazil; d Federal University of Grande Dourados, Dourados, MS, Brazil; e Oswaldo Cruz Foundation, Campo Grande, MS, Brazil; f Department of Biostatistics and Bioinformatics, Rollins School of Public Health, Emory University, Atlanta, GA, United States; g Department of Epidemiology of Microbial Diseases, Yale School of Public Health, New Haven, CT, USA; h Instituto Gonçalo Moniz, Fundação Oswaldo Cruz, Salvador, BA, Brazil; i Johns Hopkins Bloomberg School of Public Health, Baltimore, USA; j Division of Infectious Diseases and Geographic Medicine, Stanford University, Stanford, CA, USA; k Department of Biostatistics, University of Florida, Gainesville, FL, USA; l Emerging Pathogens Institute, University of Florida, Gainesville, FL, USA; m DataHealth Lab, Institut de Recerca Sant Pau (IR SANT PAU), Barcelona, Spain; n Pulmonary Division, Faculty of Medicine, Heart Institute, Hospital das Clinicas da Faculdade de Medicina da Universidade de Sao Paulo, Sao Paulo, Brazil

**Keywords:** Dengue, Vaccine, TAK-003, Symptomatic, Hospitalised

## Abstract

**Background:**

Dengue is a growing global health threat. Our initial evaluation of the TAK-003 dengue vaccine in adolescents during the 2024 outbreak in São Paulo State, Brazil, showed short-term protection but suggested possible effectiveness waning after 90-days of the first dose, and had limited power to estimate second-dose effectiveness against hospitalisation. We evaluated 1-year vaccine effectiveness against symptomatic, virologically confirmed dengue and dengue hospitalisation in adolescents during 2024–2025, when DENV-1 and DENV-2 predominated in 2024 followed by increasing DENV-3 transmission in 2025.

**Methods:**

We did a test-negative case–control study among adolescents aged 10–15 years in São Paulo State between Feb 20, 2024, and Dec 31, 2025. We extracted individual-level information on demographic characteristics, comorbidities, dengue testing, dengue vaccination, hospitalisation, and death during the study period from the national surveillance databases for dengue (SINAN-Dengue; compulsory notification) and the São Paulo State Secretary of Health vaccination registry (Rede Nacional de Dados em Saúde) on Feb 4, 2026. Cases were defined as acute febrile illness episodes with virologically confirmed dengue, as detected by NS1 antigen or RT-PCR testing within 5 days of symptom onset. Controls were test-negative acute febrile illness episodes. Surveillance data were linked to the state vaccination registry. Vaccine effectiveness after one or two TAK-003 doses was estimated with mixed-effects logistic regression.

**Findings:**

We analysed 166,390 tests: 65,365 from cases and 101,025 from controls. Vaccine effectiveness against symptomatic dengue was 59.2% (95% CI 56.1–62.0) after one dose and 73.1% (69.1–76.6) after two doses. Against dengue hospitalisation, effectiveness was 74.6% (62.7–82.7) after one dose and 87.9% (73.0–94.6) after two doses. One-dose protection against symptomatic disease showed no evidence of waning over 1-year follow-up: effectiveness was 73.8% (64.7–80.6) at 270–364 days and 76.8% (59.3–86.8) at 365 days or more. Two-dose protection remained stable to 1 year (74.6%, 95% CI 59.0–84.2, at 270–365 days). Findings were robust in sensitivity analyses and comparable during increased DENV-3 circulation.

**Interpretation:**

Our findings show that TAK-003 demonstrated sustained 1-year protection against symptomatic dengue and dengue hospitalisation in adolescents during a large outbreak. Notably, a single dose showed sustained effectiveness without evidence of waning over 1 year, generating an important hypothesis regarding dose reduction that warrants continued long-term observational evaluation. A second dose provided optimal protection, particularly against hospitalisation. These findings reinforce the importance of dengue vaccination as a public health measure.

**Funding:**

National Council for Scientific and Technological Development.

**Research in context**

**Evidence before this study**

We searched PubMed and Embase from database inception to February 2026 using the terms “TAK-003", “dengue vaccine”, “effectiveness”, and “real-world” or “observational”. Prior to our study, evidence on TAK-003 effectiveness came primarily from the phase III DEN-321 trial (Tetravalent Dengue Vaccine Efficacy [TIDES] trial), which demonstrated 80.2% efficacy against virologically confirmed dengue and 90.4% against hospitalisation over 4.5 years of follow-up. Real-world evidence was limited to our initial 6-month evaluation from São Paulo State and ecological studies from Paraná State, Brazil. No study had evaluated TAK-003 effectiveness beyond one year of follow-up in a real-world setting, nor during a period of increasing DENV-3 circulation.

**Added value of this study**

This test-negative case–control study provides the first 1-year follow-up evaluation of TAK-003 effectiveness in a real-world setting, encompassing over 166,390 dengue tests among adolescents aged 10–15 years in São Paulo State, Brazil. We found sustained vaccine effectiveness of 59.2% (one dose) and 73.1% (two doses) against symptomatic dengue, and 74.6% (one dose) and 87.9% (two doses) against hospitalisation, during a period characterised by co-circulation of DENV-1, DENV-2, and increasing DENV-3. The study demonstrates a clear dose-response relationship, with two doses providing substantially greater protection than one dose across all outcomes and time periods.

**Implications of all the available evidence**

Our findings suggest that the two-dose TAK-003 schedule provides robust and sustained protection against symptomatic dengue and hospitalisation in adolescents over one year of follow-up, even during a period of serotype transition. The observation that a single dose also conferred meaningful protection warrants further investigation through appropriately designed studies, as has been the case historically for other vaccines where dose-reduction policies were informed by accumulating real-world evidence. These data support the continued implementation of TAK-003 vaccination programmes and highlight the importance of ongoing post-marketing surveillance to monitor long-term effectiveness across different epidemiological contexts.

## Introduction

Dengue is an arboviral disease transmitted by Aedes mosquitoes^1^ that poses a growing global health threat, with over half the population seropositive in endemic regions of Asia and the Americas.^2^^,^^3^ Climate-driven geographic expansion has increased transmission worldwide, a trend projected to intensify in the coming decades. Adolescents and young adults bear a disproportionate burden in hyperendemic settings,^3^ reflecting the high force of infection and the concentration of secondary infections in this age group, which are associated with more severe clinical outcomes.^4^ Accordingly, the WHO has recommended TAK-003 vaccination for children aged 6–16 years in high-burden areas.^5^ Brazil exemplifies the public health challenge posed by dengue: over the past 25 years, dengue has caused nearly 18 million cases and approximately 17,000 deaths,^6^^,^^7^ culminating in the country's worst recorded epidemic in 2024 with more than 6000 deaths,^7^ followed by sustained transmission into 2025 with notable shifts in circulating serotypes.

To date, three tetravalent live-attenuated dengue vaccine candidates have undergone phase 3 evaluation: CYD-TDV (Dengvaxia; Sanofi Pasteur, Lyon, France),^8^ TAK-003 (Qdenga; Takeda Pharmaceuticals, Tokyo, Japan),^9^ and the Butantan-Dengue Vaccine (Butantan-DV; Instituto Butantan, São Paulo, Brazil).^10^ CYD-TDV, the first to reach licensure, demonstrated initial efficacy against both mild and severe virologically confirmed dengue in its pivotal trial.^8^ Subsequent long-term safety monitoring, however, identified an elevated risk of severe dengue-related adverse events among vaccinees younger than 9 years, particularly beyond 3 years post-vaccination,^11^ as well as among individuals who were dengue-seronegative at the time of immunisation.^12^ These safety signals were subsequently confirmed by post-licensure evaluations in Cebu, Philippines,^13^^,^^14^ and Paraná State, Brazil.^15^^,^^16^

A national vaccination campaign with TAK-003 was launched during the 2024 outbreak, prioritising adolescents aged 10–14 years, with the second dose recommended 90 days after the first.^17^ In our previous study, we evaluated the short-term effectiveness of TAK-003 during the 2024 outbreak in São Paulo state and found vaccine effectiveness of 50.2% for the first dose and 61.7% for the second dose against symptomatic dengue, and 67.5% for the first dose against hospitalisation.^18^ However, we observed a potential decline in first-dose effectiveness after 90 days and were unable to estimate second-dose effectiveness against hospitalisation owing to the limited number of events. In this study, we extend the follow-up to an additional 1 year to evaluate the durability of protection provided by one and two doses of TAK-003 against symptomatic, virologically confirmed dengue and hospitalisation with dengue among adolescents aged 10–15 years in São Paulo State during the 2024 outbreak and subsequent sustained dengue transmission in 2025, and to assess vaccine performance in the context of shifting serotype dynamics. DENV-1 and DENV-2 were the predominant circulating serotypes during 2024, while DENV-3, which had been largely absent from São Paulo for over a decade, re-emerged and became increasingly prevalent during 2025.

## Methods

### Study setting

This test-negative, case–control study used surveillance data collected in the state of São Paulo, Brazil, from Feb 20, 2024, to Dec 31, 2025. Data were extracted on Feb 4, 2026. The state has 44.4 million inhabitants, of whom approximately 2.7 million are aged 10–14 years,^19^ and contains 645 municipalities, which are divided by the infectious disease surveillance system into 28 surveillance areas (Grupo de Vigilância Epidemiológica [GVE]). On Feb 20, 2024, the Secretary of Health of the state of São Paulo initiated a dengue vaccination campaign targeting adolescents aged 10–14 years, involving the administration of a two-dose regimen of the TAK-003 vaccine (Qdenga; Takeda Pharmaceuticals) with a 3-month interval between doses. Because of the scarcity of doses, 392 municipalities (2.4 million aged 10–14 years) with high incidence of the disease were prioritised to receive the vaccine up to Feb 28, 2025, when the TAK-003 vaccine was offered to the entire state. As of Dec 31, 2025, 2,159,521 doses (1,417,538 first doses and 741,883 s doses) of TAK-003 had been administered to the target population in the 645 municipalities. The majority of these vaccines were still in the prioritised municipalities: 1,350,605 (95%) first doses and 722,358 (97%) second doses.

### Study design and ethics

The study aimed to evaluate the effectiveness of the TAK-003 dengue vaccine in reducing the odds of symptomatic, virologically confirmed dengue, as defined by the presence of the NS1 antigen or by RT-PCR, in adolescents aged 10–15 years. In this approach, individuals who test positive (referred to as test-positive cases) or negative (test-negative controls) are identified from a pool of individuals undergoing diagnostic testing for dengue.^20^ Because the study population comprises tested patients, all participants share similar health-care-seeking motivation and access to clinical testing. The test-negative design has been widely used to estimate the effectiveness of influenza^21^ and COVID-19^22^ vaccines.

This study was approved by the Ethical Committee for Research of the Federal University of Mato Grosso do Sul (CAAE: 83214124.4.0000.0021). The requirement for written informed consent was waived owing to the retrospective observational design using routinely collected de-identified surveillance data. This study was conducted in accordance with the Declaration of Helsinki.

### Participants

The study population was adolescents aged 10–15 years who had a residential address in the prioritised municipalities in São Paulo State across the entire study period or in any municipality for tests after Mar 1 2025; underwent NS1 or RT-PCR testing for dengue during the study period due to clinical suspicion of dengue and fever; and had complete information, which was consistent between data sources, on age, sex, residence, and the status and dates of dengue vaccination and testing. We expanded the age inclusion range from 10–14 to 10–15 years to capture those who were 14 in 2024 and turned 15 in 2025. Sex was determined from administrative records in the national surveillance databases and was included as a covariate in the adjusted models. Gender was not assessed as this information is not collected in the surveillance systems.

### Data sources

We extracted individual-level information on demographic characteristics, comorbidities, dengue testing, dengue vaccination, hospitalisation, and death during the study period from the national surveillance databases for dengue (SINAN-Dengue) and the São Paulo State Secretary of Health vaccination registry (retrieved from the National Health Data Network, Rede Nacional de Dados em Saúde), on Feb 4, 2026. In Brazil, it is compulsory to notify the SINAN-Dengue system, which covers both the public and private health sectors, of people with suspected dengue, dengue test results, and hospital admissions and deaths relating to dengue. The information technology bureau of the São Paulo State Government linked individual-level records from the two databases using the name of the adolescent, their date of birth, and the name of their mother, and provided anonymised datasets. During the study period, national guidelines recommended laboratory confirmation for all severe or hospitalised suspected cases, all pregnant women when clinically indicated, and a representative sample of mild suspected cases to monitor circulating serotypes. However, testing was performed at the discretion of the attending physician based on clinical presentation and local assay availability.

### Cases and controls

We selected cases as individuals from the study population who had a clinical suspicion of dengue and fever (i.e., acute febrile illness; defined as sudden onset of fever lasting ≤7 days, accompanied by non-specific symptoms such as headache, myalgia, rash, or retro-orbital pain, in a dengue-endemic area or during an outbreak)^23^ and either a positive NS1 antigen test or a positive dengue RT-PCR test collected within 5 days after symptom onset (Figure S1 in Appendix, p2).^24^ We selected controls as individuals from the study population who had a clinical suspicion of dengue and fever (i.e., acute febrile illness), a negative NS1 antigen test or a negative dengue RT-PCR test collected within 5 days after symptom onset, and no positive NS1 antigen or dengue RT-PCR test result in the following 14 days. If IgM-based tests were available, we excluded any NS1 antigen or dengue RT-PCR tests that yielded results discordant with IgM-based tests collected between the sixth and tenth days after symptom onset (Table S1 in Appendix, p4). We considered RT-PCR and NS1 antigen tests sufficiently comparable for case ascertainment under our inclusion criteria. This assumption is supported by the high diagnostic performance of NS1 antigen testing during the early symptomatic phase: within 5 days of symptom onset, reported sensitivity is approximately 83–94% and specificity 97–99%, while RT-PCR has reported sensitivity of 90–95% and specificity approaching 100%.^25^ For the analysis of vaccine effectiveness against dengue-related hospitalisation, several options for test-negative controls have been proposed.^20^^,^^21^ The main role of controls is to represent vaccine uptake in the source population, and we chose a priori to use a combination of community and hospitalised syndromic controls, as they better reflect vaccination status in the São Paulo state setting. Using syndromic controls also helps to minimise testing bias because asymptomatic individuals tested are often from special groups. Therefore, in the analysis of dengue-related hospitalisation, controls were symptomatic individuals, coming from the community or hospitalised, who tested negative, as for the analysis of protection against virologically confirmed dengue.

### Outcomes and exposure status

The primary outcome was vaccine effectiveness against symptomatic, virologically confirmed dengue, and the secondary outcome was vaccine effectiveness against hospitalisation with virologically confirmed dengue. We analysed vaccine effectiveness during the following time periods after the first dose of TAK-003: 0–7 days (bias indicator period), 8–13 days, 14–27 days, 28–59 days, 60–89 days, 90–179 days, 180–269 days, 270–364 days, and 365 or more days; and after the second dose: 0–27 days, 28–59 days, 60–89 days, 90–179 days, 180–269 days, and 270–365 days. As in previous test-negative studies, we evaluated vaccine effectiveness at 0–7 days after the first dose to test for potential bias,^24^ given that the vaccine probably has no or little effectiveness during this period.^26^

### Data analysis

Descriptive statistics were summarised as mean (SD) or median (IQR) for continuous or discrete variables and n (%) for categorical variables. We used mixed-effects logistic regression models, with random intercepts by surveillance areas (GVE) of residence to account for the regional clustering across municipalities, to estimate the adjusted odds ratio (aOR) of vaccination among cases and controls. We estimated vaccine effectiveness as 100 × (1 – aOR) under the assumptions of a test-negative design for both symptomatic dengue and hospitalisation.^21^ We included the following as covariates in the adjusted model: age (in years), sex, self-reported skin colour or race, having a chronic comorbidity, municipality of residence being previously classified as priority or not, and calendar date of symptoms onset (modelled with a natural spline with 5 df). We defined the best number of df by fitting models from linear to 8 dfs in a natural spline and comparing them with likelihood-ratio tests. A missingness indicator was incorporated to address missing information on self-reported skin colour or race, since our previous analysis using multiple imputation of missing self-reported skin colour or race showed similar results to using missing indicator in this scenario.^18^ The reference group for all vaccine effectiveness analyses was unvaccinated individuals.

We ran five sensitivity analyses. First, we included results from the NS1 antigen and RT-PCR tests collected within 7 days after symptom onset, compared with 5 days after symptom onset in our primary analysis. Second, we considered not only the results of the NS1 antigen and RT-PCR tests to define cases and controls, but also the results of IgM-based tests: when only one type of test was available, we classified the result as positive or negative on the basis of that test; and when both NS1 antigen or RT-PCR tests and IgM-based tests were available, we considered a test positive if either or both returned a positive result and negative if both returned negative results. Third, we expanded the study population to include all individuals who underwent NS1 or RT-PCR testing presenting with any symptom, regardless of the presence of fever, rather than restricting to those with fever as required in the main analysis. Fourth, we expanded the analysis to include the entire state of São Paulo, including the tests in the non-priority municipalities before March 1st 2025. Fifth, we adjusted the models for calendar time using natural splines with seven knots (Table S1 in Appendix, p4). In a post-hoc analysis, we added an interaction term between vaccination status and year of testing to the main model to assess whether vaccine effectiveness estimates differed between years.

All inclusion and exclusion criteria, the modelling approach, and the confounding adjustments applied in the main analysis were also applied in the sensitivity analyses. All analyses were conducted in R, version 4.5.1.

### Role of the funding source

The funder of the study had no role in study design, data collection, data analysis, data interpretation, or writing of the report.

## Results

**Fig. 1:**
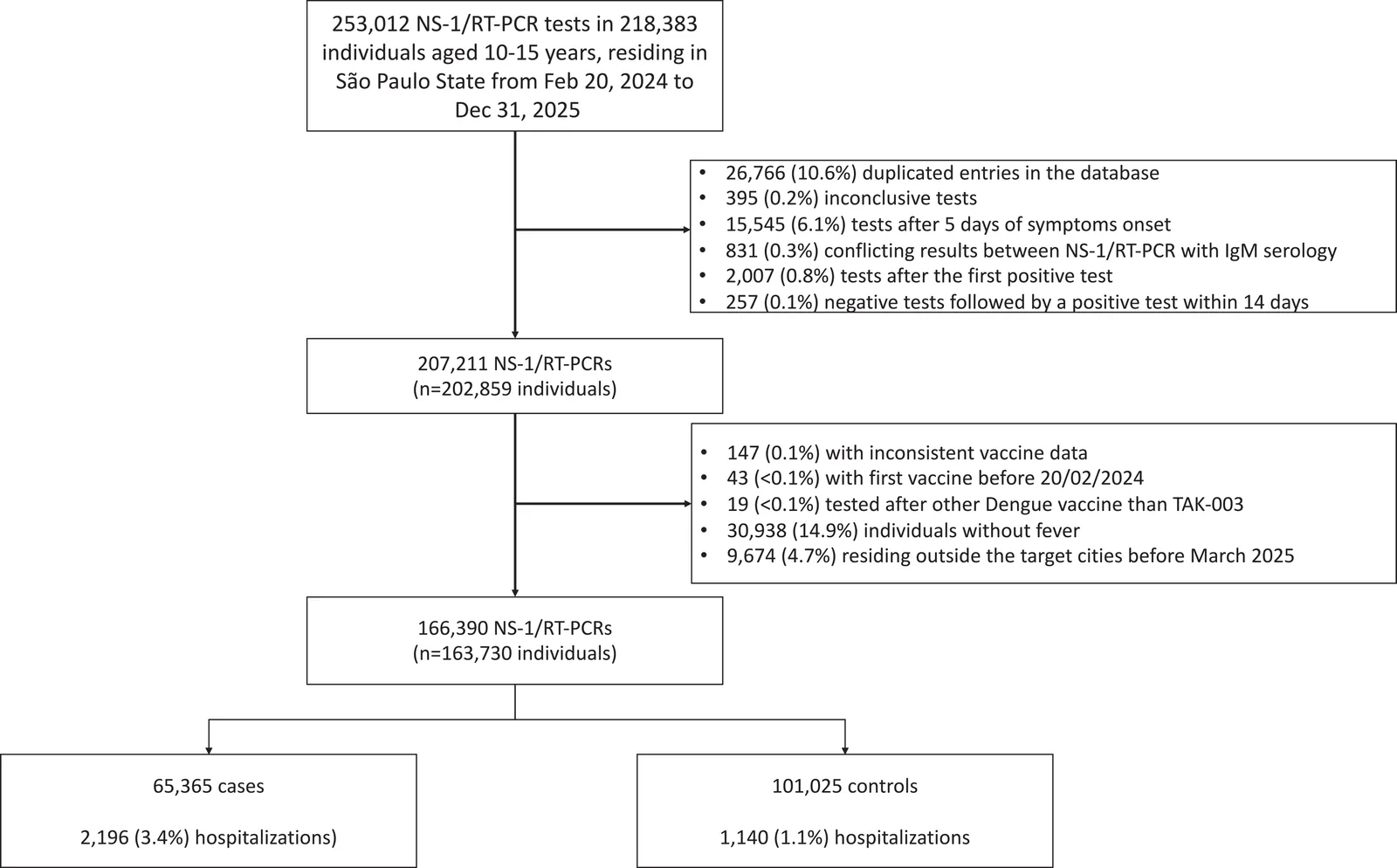
**Study flowchart.** Excluded groups (tests collected after 5 days of symptom onset, tests with conflicting NS-1/RT-PCR and IgM serology results, and tests from individuals without fever) were retained for sensitivity analyses 1, 2, and 3, respectively.

From a pool of 207,211 available NS1 antigen or RT-PCR tests, 166,390 tests were included in the analysis after applying the inclusion and exclusion criteria (Fig. 1). Of the 166,390 tests included, the majority were NS1 antigen tests: RT-PCR tests were 503 (0.5%) in the test-negative controls and 447 (0.7%) in test-positive cases. Mean age was 12.4 (SD 1.7) years in test-negative controls and 12.6 (SD 1.7) years in test-positive cases. Of the 101,025 test-negative controls, 54,485 (53.9%) were male, 1957 (1.9%) had at least one chronic comorbidity, approximately half were white, and 47,781 (47.3%) resided in São Paulo city. Of the 65,365 test-positive cases, 38,121 (58.3%) were male, 1284 (2.0%) had at least one chronic comorbidity, and 26,751 (40.9%) resided in São Paulo city (Table 1). During the study period, 440,225 suspected dengue case notifications were recorded among 384,546 adolescents aged 10–15 years in the study area. Among these notified individuals, 218,383 underwent NS1 antigen or RT-PCR testing, corresponding to a testing coverage of 57%. Of the 384,546 notified individuals, 317,077 were unvaccinated at notification, of whom 176,150 were tested for NS1 antigen or RT-PCR (56%), and 67,469 had received at least one vaccine dose before notification, of whom 42,233 were tested for NS1 antigen or RT-PCR (63%).

**Table 1:** Participant characteristics.

	Test-negative controls (N = 101,025)	Test-positive cases (N = 65,365)
Mean age, years (SD)	12.4 (1.7)	12.6 (1.7)
Age, years (n, %)		
10	17,714 (17.5%)	10,367 (15.9%)
11	17,353 (17.2%)	10,314 (15.8%)
12	16,902 (16.7%)	11,008 (16.8%)
13	16,489 (16.3%)	11,032 (16.9%)
14	16,209 (16.0%)	11,065 (16.9%)
15	16,358 (16.2%)	11,579 (17.7%)
Sex		
Female	46,540 (46.1%)	27,244 (41.7%)
Male	54,485 (53.9%)	38,121 (58.3%)
Self-reported race or skin colour		
White	51,477 (51.0%)	34,104 (52.2%)
Pardo	33,980 (33.6%)	18,148 (27.8%)
Black	5898 (5.8%)	3129 (4.8%)
Asian	1017 (1.0%)	531 (0.8%)
Indigenous	181 (0.2%)	151 (0.2%)
Missing	8472 (8.4%)	9302 (14.2%)
Residence in São Paulo capital	47,781 (47.3%)	26,751 (40.9%)
Reported number of comorbidities		
0	99,068 (98.1%)	64,081 (98.0%)
1–2	1957 (1.9%)	1284 (2.0%)
Hospitalisation	1140 (1.1%)	2196 (3.4%)
Death	34 (<0.1%)	13 (<0.1%)
Type of test		
NS1 antigen	100,522 (99.5%)	64,918 (99.3%)
RT-PCR	503 (0.5%)	447 (0.7%)
Interval between symptom onset and NS1 antigen or RT-PCR testing, days, median, [IQR]	2 (1–3)	2 (1–3)
Vaccination status		
Unvaccinated	90,590 (89.7%)	64,133 (98.1%)
Single dose	6399 (6.3%)	1008 (1.5%)
Two doses	4036 (4.0%)	224 (0.3%)
Interval between first dose and symptom onset, days, median, [IQR]	74 (29–179)	18 (6–62)
Interval between second dose and symptom onset, days, median, [IQR]	173 (79–256)	157 (60–226)

Data are mean (SD), n (%), or median (IQR).

Among controls, 90,590 (89.7%) were unvaccinated, 6399 (6.3%) had received one dose, and 4036 (4.0%) had received two doses. Among cases, 64,133 (98.1%) were unvaccinated, 1008 (1.5%) had received one dose, and 224 (0.3%) had received two doses. Among vaccinated individuals, the median interval from the first dose to symptom onset was 74 days [IQR 29–179] for controls and 18 days [IQR 6–62] for cases; for the second dose, the interval was 173 days [IQR 79–256] for controls and 157 days [IQR 60–226] for cases. Hospitalisation occurred in 2196 (3.4%) of cases and 1140 (1.1%) of controls.

**Fig. 2:**
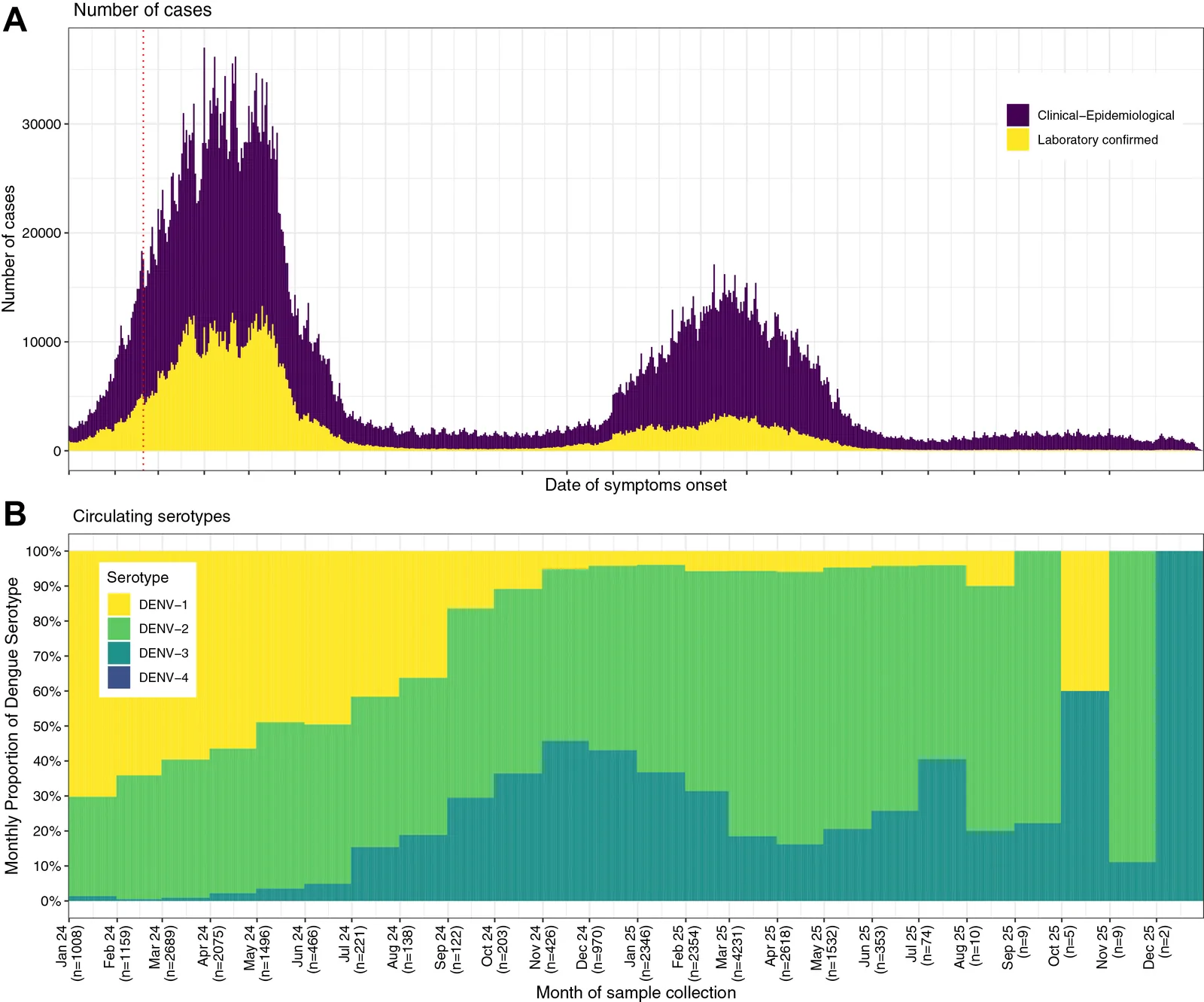
**Dengue cases and serotype distribution for the entire state of São Paulo and all age groups.** (A) Daily number of laboratory-confirmed and clinical/epidemiologically confirmed dengue cases across all ages in the São Paulo state. (B) Dengue serotype distribution over time of 24,522 specimens across all ages in the São Paulo state (there were seven cases of DENV-4).

The epidemic curve and serotype distribution patterns across all age groups in São Paulo State are shown in Fig. 2. São Paulo state experienced continued dengue transmission during the 2024–2025 period. Dengue virus serotype distribution shifted over time: while DENV-1 and DENV-2 remained the predominant serotypes during 2024, the relative prevalence of DENV-3 increased notably during the study period. Additionally, six cases of DENV-4 were detected during 2025 (one in February, four in March, and one in May).

Vaccine effectiveness against symptomatic, virologically confirmed dengue was 59.2% (95% CI 56.1–62.0) for the first dose and 73.1% (69.1–76.6) for the second dose (Table 2). Against hospitalisation with virologically confirmed dengue, vaccine effectiveness was 74.6% (62.7–82.7) for the first dose and 87.9% (73.0–94.6) for the second dose (Table 2).

**Table 2:** Vaccine effectiveness against symptomatic, virologically confirmed dengue and hospitalisation with dengue.

	Number of test-positive/test-negative	Unadjusted vaccine effectiveness (95% CI)	Adjusted vaccine effectiveness (95% CI)^a^
Symptomatic dengue (N = 166,390)			
Unvaccinated	64,133/90,590	Reference	Reference
First dose	1008/6399	77.2% (75.6–78.6)	59.2% (56.1–62)
Second dose	224/4036	91.9% (90.8–92.9)	73.1% (69.1–76.6)
Symptomatic dengue by time after first dose			
Unvaccinated	64,133/90,590	Reference	Reference
0–7 days	352/461	−11.7% (−23.2–1.6)	−2.4% (−15.8–11.6)
8–13 days	126/490	62.6% (54.4–69.3)	64.2% (56–70.8)
14–27 days	108/582	72.5% (66.2–77.6)	68.8% (61.4–74.8)
28–59 days	160/1209	80.4% (76.9–83.4)	69.2% (63.5–74)
60–89 days	73/838	87.2% (83.7–89.9)	70.2% (62–76.7)
90–179 days	92/1229	89.2% (86.6–91.2)	63.8% (55.1–70.9)
180–269 days	37/561	90.8% (87.2–93.4)	66.2% (52.3–76.1)
270–364 days	47/641	89.8% (86.3–92.4)	73.8% (64.7–80.6)
≥365 days	13/388	95.3% (91.9–97.3)	76.8% (59.3–86.8)
Symptomatic dengue by time after second dose			
0–27 days	25/369	90.4% (85.6–93.6)	72.2% (58–81.6)
28–59 days	31/416	89.1% (84.3–92.5)	66.3% (51.1–76.8)
60–89 days	21/352	91.5% (86.8–94.6)	75.8% (62.1–84.5)
90–179 days	53/945	91.9% (89.3–93.9)	76.3% (68.5–82.1)
180–269 days	68/1077	90.6% (88–92.7)	75% (67.9–80.5)
270–365 days	18/489	94.6% (91.4–96.6)	74.6% (59–84.2)
Hospitalisation with dengue			
Unvaccinated	2163/90,590	Reference	Reference
First dose	27/6399	82.4% (74.3–88)	74.6% (62.7–82.7)
Second dose	6/4036	93.7% (85.9–97.2)	87.9% (73–94.6)

In the adjusted estimates, we included as covariates age, sex, self-reported race or skin colour, presence or absence of chronic comorbidities, municipality of residence being previously classified as priority or not, and calendar time (natural spline with 5 df).

a Vaccine effectiveness estimates were obtained from mixed logistic regression models with random intercepts for each surveillance area.

**Fig. 3:**
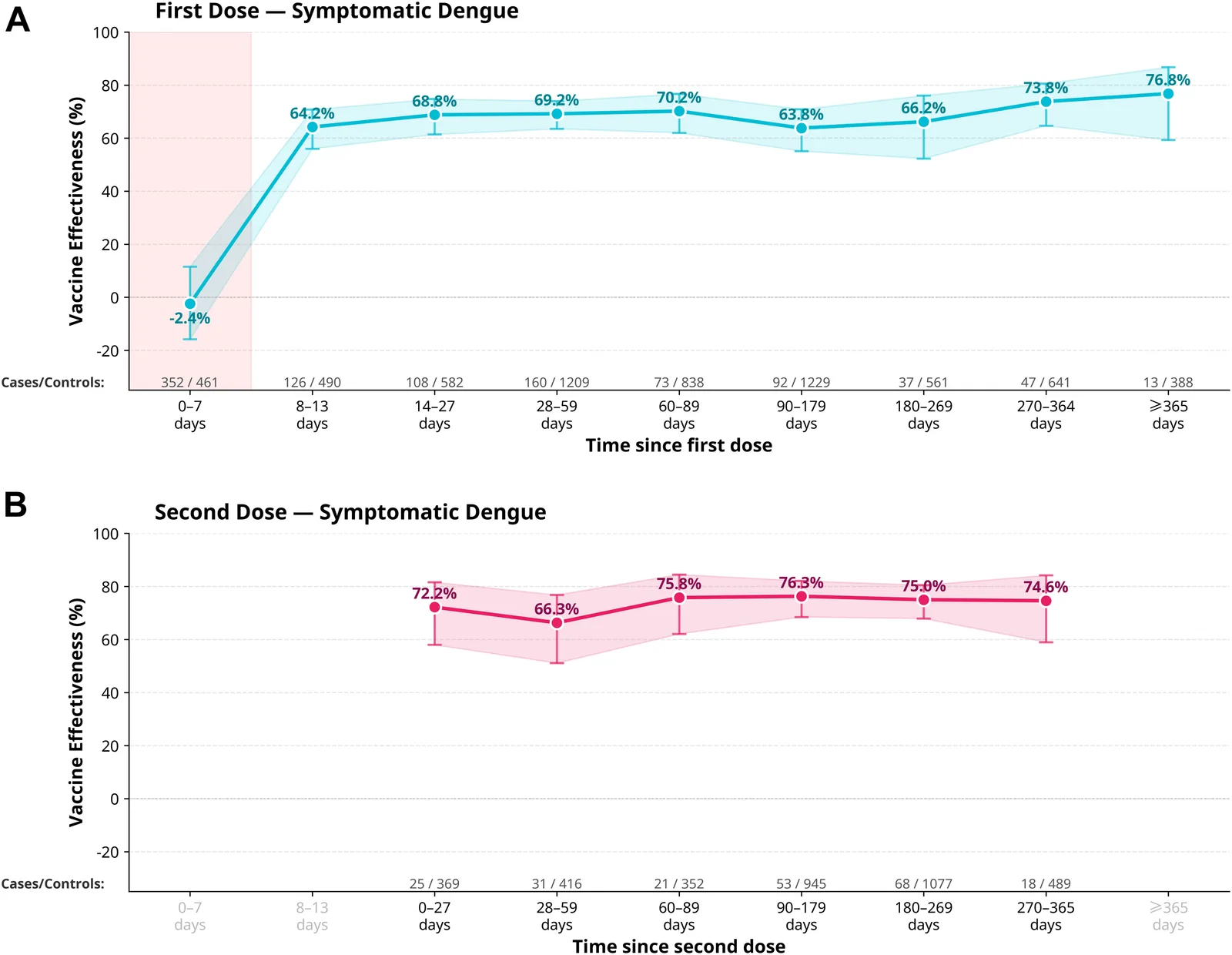
**Sensitivity analyses of adjusted vaccine effectiveness against symptomatic, virologically confirmed dengue and hospitalisation with dengue, São Paulo State, Brazil, 2024–2025.** Panel A shows adjusted vaccine effectiveness (aVE) estimates by time interval since the first dose of TAK-003 (0–7, 8–13, 14–27, 28–59, 60–89, 90–179, 180–269, 270–364, and ≥365 days). Panel B shows aVE estimates by time interval since the second dose (0–27, 28–59, 60–89, 90–179, 180–269, and 270–365 days), plotted on the same axes for comparability. Point estimates are shown as circles connected by a solid line, with vertical bars representing 95% confidence intervals. The shaded area depicts the 95% confidence band across time intervals. In Panel A, the red-shaded region (0–7 days after the first dose) indicates the bias indicator period, during which vaccine effectiveness is expected to be null owing to insufficient time for immune response development. The number of cases and controls for each time interval is shown below the x-axis.

When analysing different time periods after the first dose, we observed no vaccine protection against symptomatic dengue during the bias indicator period of 0–7 days (−2.4% 95% CI −15.8 to 11.6; p = 0.75), followed by substantial protection starting at 8–13 days (64.2% 95% CI 56.0–70.8). Protection was maintained at high levels throughout the follow-up: 68.8% (61.4–74.8) at 14–27 days, 69.2% (63.5–74.0) at 28–59 days, 70.2% (62.0–76.7) at 60–89 days, 63.8% (55.1–70.9) at 90–179 days, 66.2% (52.3–76.1) at 180–269 days, 73.8% (64.7–80.6) at 270–364 days, and 76.8% (59.3–86.8) at 365 or more days after the first dose (Table 2, Fig. 3). Notably, there was no evidence of waning effectiveness; instead, the point estimates of vaccine effectiveness remained stable over time.

For the second dose, vaccine protection against symptomatic dengue was observed from the earliest time period evaluated (72.2% [58.0–81.6] at 0–27 days) and remained stable throughout the follow-up: 66.3% (51.1–76.8) at 28–59 days, 75.8% (62.1–84.5) at 60–89 days, 76.3% (68.5–82.1) at 90–179 days, 75.0% (67.9–80.5) at 180–269 days, and 74.6% (59.0–84.2) at 270–365 days (Table 2, Fig. 3).

Serotype information was available for only 845 test-positive cases (1.3%), limiting serotype-specific inference. Among these, 453 were DENV-2 (53.6%), 283 DENV-1 (33.5%), and 109 DENV-3 (12.9%). Among those vaccinated, only 25 test-positive cases had serotype information available: 10 DENV-2 cases, all after the first dose; 10 DENV-1 cases, of which 8 occurred after the first dose; and 5 DENV-3 cases, of which 4 occurred after the first dose.

Overall, all sensitivity analyses showed similar results to the main analysis (Figure S2; Tables S2 and S3 in Appendix, pp3, 5 and 6). When including tests up to 7 days after symptom onset, the vaccine effectiveness was 58.6% (55.6–61.4) for the first dose and 72.9% (68.9–76.3) for the second dose against symptomatic dengue. When combining IgM-based tests, we found 53.4% (50.3–56.3) for the first dose and 68.1% (64.0–71.7) for the second dose. When including patients with any symptom, the values were 57.0% (54.1–59.7) and 71.5% (67.6–74.9), respectively. When expanding to the entire state of São Paulo, the vaccine effectiveness was 59.8% (56.8–62.6) for the first dose and 73.9% (70.0–77.3) for the second dose. When modelling epidemic time with a more flexible spline, the values were 58.8% (55.7–61.6) and 72.5% (68.4–76.1). When excluding early-post vaccination periods, vaccine effectiveness for the first dose was 68.3% (65.5–70.9) after 7 days and 69% (66–71.8) after 13 days (Table S4 in Appendix, p7). Vaccine effectiveness remained stable across calendar periods characterised by increasing DENV-3 circulation at the population level (Table S5 in Appendix, p8).

Regarding hospitalisation, all sensitivity analyses yielded consistent results: first-dose effectiveness ranged from 67.3% to 75.1%, and second-dose effectiveness ranged from 82.3% to 88.1% across all analyses (Figure S2 in Appendix, p3).

## Discussion

In this study of adolescents aged 10–15 years conducted during the 2024–2025 dengue epidemic in São Paulo State, we estimated TAK-003 vaccine effectiveness against symptomatic dengue of 59% for the first dose and 73% for the second dose. We further estimated vaccine effectiveness against hospitalisation of 75% for the first dose and 88% for the second dose. Building upon our prior analysis, a central finding is the absence of waning effectiveness after a single dose over 1 year of follow-up, with vaccine effectiveness remaining above 60% at all time points and reaching 77% at 365 or more days. Furthermore, we provide the first real-world estimates of second-dose TAK-003 effectiveness against hospitalisation, demonstrating high and sustained protection. These findings were maintained in sensitivity analyses and were observed despite the increasing circulation of DENV-3 in the São Paulo State.

The durability of single-dose protection observed in this study contrasts with the preliminary decline we reported in our initial analysis, where vaccine effectiveness decreased to 49.7% (95% CI 30.4–63.6) after 90 days from the first dose.^18^ With the extended follow-up and larger sample size, we now demonstrate that first-dose protection is maintained at high levels through 1 year, with no evidence of waning. This discrepancy likely reflects the limited power and shorter observation period of our initial study, where the apparent decline may have been influenced by confounding factors related to the timing of vaccination during the peak of the epidemic. The current findings are reassuring and suggest that a single dose of TAK-003 provides sustained effectiveness against symptomatic dengue over 1 year.

The overall vaccine effectiveness estimates in this study are higher than those reported in our initial analysis (59%, 95% CI 56–62 vs 50%, 95% CI 45–55 for the first dose; 73%, 95% CI 69–77 vs 62%, 95% CI 40–76 for the second dose against symptomatic dengue).^18^ This improvement likely reflects the accumulation of more events over the extended follow-up period, reducing the influence of early epidemic dynamics characterised by a high force of infection, and providing more stable estimates of true vaccine protection. The overall VE estimate is influenced by the proportion of cases occurring during the 0–7 day bias indicator period, when no protection is expected. In the present analysis, 35% of first-dose cases occurred in this window compared with 41% in our initial study, which may partly explain differences in overall point estimates between the two evaluations. Indeed, the first dose VE against symptomatic disease increases to 68.3% (65.5–70.9) if the first seven days period is excluded. Our second dose estimates against symptomatic dengue are similar to those observed in the long-term follow-up of the phase 3 trial (63.5% for ages 6–11 years and 67.7% for ages 12–16 years),^27^^,^^28^ despite differences in follow-up time and population characteristics.

The overall vaccine effectiveness remained stable during a period characterised by increasing DENV-3 circulation at the population level in São Paulo state. While DENV-1 and DENV-2 were the predominant serotypes during 2024, the relative prevalence of DENV-3 increased notably, and isolated cases of DENV-4 were detected in early 2025. The maintenance of vaccine effectiveness in this shifting serotype landscape is a positive finding, given that the phase 3 trial of TAK-003 reported relatively lower efficacy against DENV-3 (62.6%) compared with DENV-1 (73.7%) and DENV-2 (97.7%).^9^ However, individual infections among study participants were not routinely serotyped, and we cannot determine whether the maintained effectiveness reflects protection against DENV-3 specifically or continued protection against DENV-1 and DENV-2, which remained in circulation throughout the study period. Continued monitoring with serotype-specific data is warranted.

The high effectiveness of the second dose against hospitalisation (87.9%) is a notable addition to the evidence base for TAK-003. In our previous study, we were unable to estimate this outcome owing to the small number of events.^18^ The current estimate is consistent with the long-term efficacy reported in the phase 3 trial (84.1% against hospitalisation with virologically confirmed dengue),^9^ and provides important real-world confirmation of the vaccine's ability to prevent severe disease requiring hospital admission.

While a single dose demonstrated sustained effectiveness over 1 year, we emphasise that our study was not designed to compare one-dose and two-dose strategies directly. The two-dose schedule consistently showed higher effectiveness, particularly against hospitalisation (88% vs 75%), and remains the recommended regimen. These findings generate an important hypothesis regarding single-dose protection. As seen historically with the HPV,^29^^,^^30^ yellow fever,^31^ and pneumococcal conjugate vaccines,^32^^,^^33^ robust real-world observational data and long-term follow-up will be essential to evaluate whether a reduced-dose schedule could be considered by public health authorities in the future. Additionally, the vaccine could serve as a tool to mitigate the risk of disease for travellers visiting regions with high dengue transmission, given the rapid onset of protection observed after the first dose.

Our study has several methodological strengths. It represents, to our knowledge, the longest real-world follow-up evaluation of TAK-003 to date, extending the observation period to 1 year and thereby enabling assessment of the durability of vaccine-induced protection. We leveraged the surveillance system of São Paulo state, encompassing over 44 million inhabitants during two consecutive high-transmission seasons, resulting in an analytic sample of 166,390 individuals that afforded sufficient statistical power for time-stratified effectiveness analyses. The test-negative case–control design inherently adjusts for differential healthcare-seeking behaviour between vaccinated and unvaccinated individuals, a property that has been validated for dengue vaccine evaluations in prior studies, and is important in contexts where uptake/access to care and to vaccine are correlated.^20^^,^^21^ Furthermore, the bias indicator analysis provides internal validation of our approach: the absence of measurable protection during the first 7 days post-vaccination, followed by a rapid rise in effectiveness, is consistent with the expected biological timeline of immune response development and argues against substantial residual confounding.^24^

Our study has several limitations. First, baseline dengue serostatus was unavailable for this population, precluding stratified analyses by prior infection history. The observed vaccine effectiveness likely reflects a combination of vaccine-induced immunity and boosting of pre-existing natural immunity in previously exposed individuals. Based on data from a phase 3 clinical trial conducted across 15 municipalities in Brazil,^10^ approximately 41% of participants in the Southeast region had evidence of prior dengue exposure at baseline,^34^ which is substantially lower than the 72% seroprevalence observed in the TIDES trial,^35^ suggesting that boosting of pre-existing immunity may contribute less to the observed effectiveness in our population. However, since vaccination was determined by a public health campaign targeting all adolescents aged 10–14 years across São Paulo State irrespective of prior infection history, there is no plausible mechanism by which baseline serostatus would differ systematically between vaccinated and unvaccinated individuals, and it is therefore unlikely to introduce differential confounding in our estimates. Second, passive surveillance inherently underestimates the true burden of infection, as individuals with subclinical or mildly symptomatic disease may not seek care, which may limit the generalisability of our effectiveness estimates to the full spectrum of dengue illness. Third, our study estimates overall vaccine effectiveness against dengue-associated hospitalisation but does not directly assess whether vaccination reduces the probability of progression to hospitalisation among those who acquire DENV infection; a case-only analysis comparing hospitalised and non-hospitalised confirmed cases was precluded by the small number of vaccinated individuals who required hospitalisation. Fourth, diagnostic misclassification cannot be excluded. The predominant use of NS1 antigen detection rather than molecular methods introduces the possibility of false-negative results, particularly when specimens are collected beyond the first few days of illness; however, recent meta-analytic evidence indicates that the pooled diagnostic accuracy of NS1 and RT-PCR assays is broadly comparable.^25^ Fifth, the limited availability of serotyping data precluded serotype-specific effectiveness estimation, as the number of typed specimens was insufficient to generate stable estimates for individual viral lineages. Sixth, while our data show no evidence of waning effectiveness over 12 months, this observation period remains relatively short. Longer follow-up is essential to determine whether protection persists beyond 1 year and to monitor for potential late-onset safety signals, particularly among seronegative individuals, given the experience with CYD-TDV. Seventh, the test-negative design reduces bias from differential healthcare-seeking because cases and controls are drawn from the same population of symptomatic individuals who sought care, were tested, and were notified. However, residual selection bias may remain if vaccination status influenced care-seeking, testing, or reporting among symptomatic adolescents. In addition, if vaccination reduced disease severity among breakthrough infections, vaccinated cases may have been less likely to seek care or be tested, potentially affecting estimates of vaccine effectiveness against medically attended symptomatic dengue. Reassuringly, the bias-indicator analysis showed no evidence of early protection, arguing against substantial early differential testing or reporting by vaccination status. Nevertheless, smaller residual biases cannot be excluded.

We showed sustained real-world protection of the TAK-003 vaccine against symptomatic, virologically confirmed dengue and hospitalisation with dengue in adolescents over a 1-year follow-up, highlighting the importance of dengue vaccines as a public health measure. The two-dose schedule provides optimal protection, particularly against hospitalisation. While a single dose showed sustained effectiveness over 1 year, these observational findings are hypothesis-generating and warrant continued long-term real-world studies to evaluate the feasibility of alternative vaccination schedules.

## Contributors

LKM, OR, RDdO, TLD’A, RACDP, and JC designed and conceived the study. OR and TSB analysed the data. All authors interpreted the data. LKM wrote the first version of the manuscript, which was reviewed by TSB, RDdO, PVdS, ERdS, TLD’A, RACDP, NED, AIK, DATC, JRA, MDTH, OR, and JC. JC and OR supervised the study. OR and JC accessed and verified the underlying data. All authors had full access to all the data in the study and had final responsibility for the decision to submit for publication. All authors read and approved the final version of the manuscript.

## Data sharing statement

The relevant data are available in the Article and its Appendix. The raw data cannot be publicly shared owing to ethical restrictions and the presence of sensitive personal information. Researchers may request access to the raw data from the Centro de Vigilância Epidemiológica “Prof. Alexandre Vranjac”, São Paulo State Department of Health, in compliance with their policies and data use agreements; see https://saude.sp.gov.br/cve-centro-de-vigilancia-epidemiologica-prof.-alexandre-vranjac/pesquisa-cve/. Requests should be sent to dvmetodo@saude.sp.gov.br.

## Declaration of interests

JC reports advisory board participation and the receipt of consulting fees and research grants from Takeda Pharmaceuticals. MDTH reports contracts (awarded to the University of Florida) from Merck, Sharp & Dohme for research unrelated to this manuscript. All other authors declare no competing interests.
