# Supplementary material for: 1-Year follow-up evaluation of TAK-003 dengue vaccine effectiveness against symptomatic disease and hospitalisation in adolescents during the 2024–2025 outbreak in São Paulo, Brazil: a test-negative case-control study
Source: eClinicalMedicine. 2026 Aug 31;99:104181. doi: 10.1016/j.eclinm.2026.104181 (PMC13562391; doi:10.1016/j.eclinm.2026.104181)
Supplement: Supplementary file 1 [file mmc1.docx]

**Supplementary appendix**

Supplement to: Mareto LK, Brumatti TS, de Oliveira RD, et al. One-year follow-up evaluation of TAK-003 dengue vaccine effectiveness against symptomatic disease and hospitalisation in adolescents during the 2024-2025 outbreak in São Paulo, Brazil: a test-negative study

**Table of Contents**

Figure S1. Distribution of NS1/RT-PCR dengue tests during the study period stratified by test result and vaccination status. (p. 2)

Figure S2. Adjusted vaccine effectiveness of TAK-003 against symptomatic dengue and hospitalisation, by sensitivity analysis, São Paulo State, Brazil, 2024–2025. (p. 3)

Table S1. Test-positive and test-negative definitions for the main and sensitivity analyses. (p. 4)

Table S2. Vaccine effectiveness against symptomatic dengue by time after first dose (sensitivity analyses). (p. 5)

Table S3. Vaccine effectiveness against symptomatic dengue by time after second dose – sensitivity analyses. (p. 6)

Table S4: Vaccine effectiveness against symptomatic dengue for the first dose, excluding early post-vaccination periods (“per protocol like analysis”) – sensitivity analyses

Table S5. Vaccine effectiveness against symptomatic dengue by calendar period, São Paulo State, Brazil, 2024–2025. (p. 7)

**Figure S1.** Distribution of NS1/RT-PCR dengue tests during the study period stratified by test result and vaccination status.

NS-1 denotes NS-1 antigen test; RT-PCR Reverse Transcription Polymerase Chain Reaction.

**Figure S2. Adjusted vaccine effectiveness of TAK-003 against symptomatic dengue and hospitalisation, by sensitivity analysis, São Paulo State, Brazil, 2024–2025**

Forest plot showing adjusted vaccine effectiveness (aVE) point estimates and 95% confidence intervals for each sensitivity analysis, stratified by outcome (symptomatic dengue and hospitalisation) and dose (first and second). Six analyses are presented: main analysis; restriction to NS1/RT-PCR tests performed within 7 days of symptom onset; inclusion of IgM-based tests; inclusion of any symptom as the index date; expansion to the entire São Paulo State (including tests from non-priority municipalities before March 1st 2025); and epidemic time modelled with natural cubic splines with 7 degrees of freedom. The number of cases and controls, dose, and aVE with 95% CI are shown to the left of each estimate. Each analysis is represented by a distinct colour.

**Table S1. Test-positive and test-negative definitions for the main and sensitivity analyses.**

|  |  | **Symptom** | **NS1 antigen / Dengue RT-PCR** | **Dengue IgM** | **Comment** |
| --- | --- | --- | --- | --- | --- |
| Main analysis | Test negative | At least fever | Negative  (0-5 days from symptoms) | Negative  (6-10 days from symptoms) or Not done | When more than 1 test was available, they must be negative |
|  | Test positive | At least fever | Positive  (0-5 days from symptoms) | Positive  (6-10 days from symptoms) or Not done | When more than 1 test was available, they must be positive |
| First sensitivity analysis (Up to 7 days from symptoms) | Test negative | At least fever | Negative  (0-7 days from symptoms) | Negative  (6-10 days from symptoms) or Not done | When more than 1 test was available, they must be negative |
|  | Test positive | At least fever | Positive  (0-7 days from symptoms) | Positive  (6-10 days from symptoms) or Not done | When more than 1 test was available, they must be positive |
| Second sensitivity analysis (Together NS1/RT-PCR and IgM) | Test negative | At least fever | Negative  (0-5 days from symptoms) or Not done | Negative  (6-10 days from symptoms) or Not done | When more than 1 test was available, they must be negative |
|  | Test positive | At least fever | Positive  (0-5 days from symptoms) or Not done or  Negative if IgM positive | Positive  (6-10 days from symptoms) or Not done or Negative if NS1/RT-PCR positive | When more than 1 test was available, at least one they must be positive.  This analysis considers a case (i.e., dengue) a NS1/RT-PCR test-positive even if a negative IgM was available (and vice versa), considering the high specificity of the test. |
| Third sensitivity analysis (any symptom) | Test negative | At least fever | Negative  (0-5 days from symptoms) | Negative  (6-10 days from symptoms) or Not done | When more than 1 test was available, they must be negative |
|  | Test positive | At least fever | Positive  (0-5 days from symptoms) | Positive  (6-10 days from symptoms) or Not done | When more than 1 test was available, they must be positive |
| Fourth sensitivity analysis (calendar time adjustment | Test negative | At least fever | Negative  (0-5 days from symptoms) | Negative  (6-10 days from symptoms) or Not done | Same test-negative of main analysis, difference on adjustment |
|  | Test positive | At least fever | Positive  (0-5 days from symptoms) | Positive  (6-10 days from symptoms) or Not done | Same test-positive of main analysis, difference on adjustment |

**Table S2: Vaccine effectiveness against symptomatic dengue by time after first dose (sensitivity analyses)**

| Time period | Main analysis VE (95% CI) | NS1/RT-PCR up to 7 days VE (95% CI) | Includes IgM VE (95% CI) | Any symptom VE (95% CI) | Epidemic time ns(df=7) VE (95% CI) |
| --- | --- | --- | --- | --- | --- |
| 0–7 days | −2·4%  (−15·8 to 11·6) | −2·8%  (−15·8 to 10·9) | −4·0%  (−16·6 to 9·6) | −4·9%  (−17·0 to 8·3) | −1·1%  (−14·7 to 12·8) |
| 8–13 days | 64·2%  (56·0–70·8) | 64·2%  (56·1–70·7) | 57·4%  (48·6–64·6) | 63·0%  (55·5–69·3) | 65·3%  (57·5–71·8) |
| 14–27 days | 68·8%  (61·4–74·8) | 67·6%  (60·1–73·7) | 59·0%  (50·5–66·0) | 66·0%  (58·9–71·9) | 70·9%  (64·0–76·5) |
| 28–59 days | 69·2%  (63·5–74·0) | 68·5%  (62·8–73·4) | 63·2%  (57·2–68·3) | 67·2%  (61·8–71·8) | 68·3%  (62·4–73·3) |
| 60–89 days | 70·2%  (62·0–76·7) | 69·5%  (61·3–76·0) | 64·1%  (55·8–70·9) | 66·8%  (58·9–73·2) | 66·8%  (57·6–74·0) |
| 90–179 days | 63·8%  (55·1–70·9) | 65·1%  (56·7–71·9) | 56·3%  (47·5–63·6) | 60·7%  (52·2–67·7) | 59·8%  (50·0–67·7) |
| 180–269 days | 66·2%  (52·3–76·1) | 67·3%  (53·9–76·8) | 55·0%  (40·7–65·9) | 60·8%  (46·8–71·1) | 69·9%  (57·5–78·6) |
| 270–364 days | 73·8%  (64·7–80·6) | 73·8%  (64·7–80·6) | 73·4%  (64·8–79·9) | 72·7%  (64·0–79·3) | 74·3%  (65·3–81·0) |
| ≥365 days | 76·8%  (59·3–86·8) | 75·4%  (57·7–85·7) | 73·3%  (56·7–83·5) | 74·6%  (57·8–84·7) | 74·0%  (54·5–85·2) |

Vaccine effectiveness estimates were obtained from mixed logistic regression models with random intercepts for each surveillance area, adjusted by age, sex, self-reported race or skin colour, presence or absence of chronic comorbidities, municipality of residence being previously classified as priority or not, and calendar time (natural spline with 5 df) (except the sensitivity analysis of epidemic time parametrization).

**Table S3: Vaccine effectiveness against symptomatic dengue by time after second dose – sensitivity analyses**

| Time period | Main analysis VE (95% CI) | NS1/RT-PCR up to 7 days VE (95% CI) | Includes IgM VE (95% CI) | Any symptom VE (95% CI) | Epidemic time ns(df=7) VE (95% CI) |
| --- | --- | --- | --- | --- | --- |
| 0–27 days | 72·2%  (58·0–81·6) | 72·0%  (58·0–81·3) | 61·7%  (46·7–72·4) | 71·7%  (58·2–80·8) | 70·7%  (55·7–80·6) |
| 28–59 days | 66·3%  (51·1–76·8) | 67·3%  (52·6–77·5) | 68·5%  (55·1–78·0) | 63·8%  (49·3–74·1) | 64·6%  (48·6–75·6) |
| 60–89 days | 75·8%  (62·1–84·5) | 75·1%  (61·4–83·9) | 74·2%  (61·3–82·8) | 76·8%  (64·4–84·8) | 75·2%  (61·2–83·9) |
| 90–179 days | 76·3%  (68·5–82·1) | 76·3%  (68·7–82·1) | 66·4%  (57·9–73·1) | 74·2%  (66·7–80·1) | 77·2%  (69·7–82·8) |
| 180–269 days | 75·0%  (67·9–80·5) | 74·0%  (66·9–79·5) | 72·7%  (65·9–78·2) | 72·7%  (65·9–78·2) | 73·8%  (66·4–79·6) |
| 270–365 days | 74·6%  (59·0–84·2) | 75·1%  (59·9–84·6) | 67·7%  (52·1–78·2) | 77·9%  (64·5–86·3) | 70·4%  (52·2–81·6) |

Vaccine effectiveness estimates were obtained from mixed logistic regression models with random intercepts for each surveillance area, adjusted by age, sex, self-reported race or skin colour, presence or absence of chronic comorbidities, municipality of residence being previously classified as priority or not, and calendar time (natural spline with 5 df) (except the sensitivity analysis of epidemic time parametrization).

**Table S4:** Vaccine effectiveness against symptomatic dengue for the first dose, excluding early post-vaccination periods (“per protocol like analysis”) – sensitivity analyses

|  | Number of test-positive / test-negative | Unadjusted vaccine effectiveness  (95% CI) | Adjusted vaccine effectiveness  (95% CI) |
| --- | --- | --- | --- |
| Main analysis | 1008 / 6399 | 77.2% (78.6-75.6) | 59·2% (56·1–62·0) |
| Per-protocol analysis after 7 days of first dose | 656 / 5938 | 84% (82.7 - 85.3) | 68.3% (65.5 - 70.9) |
| Per-protocol analysis after 13 days of first dose | 530 / 5448 | 85.9% (84.6 - 87.2) | 69% (66 - 71.8) |

Vaccine effectiveness estimates were obtained from mixed logistic regression models with random intercepts for each surveillance area, adjusted by age, sex, self-reported race or skin colour, presence or absence of chronic comorbidities, municipality of residence being previously classified as priority or not, and calendar time (natural spline with 5 df)

**Table S5.** Crude and Adjusted vaccine effectiveness for VCD stratified by year of testing*

|  | Year | Time from vaccination to symptoms onset, days  (median, IQR) | Test positive/Test negative | Crude  VE (95% CI) | Adjusted  VE (95% CI) |
| --- | --- | --- | --- | --- | --- |
| Unvaccinated | 2024 |  | 53083/56299 | Reference | Reference |
| First | 2024 | 29 [9-66] | 690/2135 | 63.4% (59.4 - 67.1) | 50% (44.2 - 55.1) |
| Second | 2024 | 48 [25-76] | 27/326 | 91.2% (86 - 94.5) | 50% (19.3 - 69.0) |
|  |  |  |  |  |  |
| Unvaccinated | 2025 |  | 9996/30834 | Reference | Reference |
| First | 2025 | 102 [45-262] | 312/4199 | 74.9% (71.1 - 78.2) | 69.5% (64.8 - 73.6) |
| Second | 2025 | 188 [96-263] | 196/3689 | 81.3% (77.8 - 84.3) | 74.7% (69.8 - 78.8) |

P<0.0001 for the interaction term between year and vaccine effectiveness for both crude and adjusted estimates.

* To keep the comparability between years, for this analysis, only tests from the prioritized cities were analysed in both years.

Vaccine effectiveness estimates were obtained from mixed logistic regression models with random intercepts for each surveillance area, adjusted by age, sex, self-reported race or skin colour, presence or absence of chronic comorbidities, municipality of residence being previously classified as priority or not, and calendar time (natural spline with 5 df)
